# The Clinical Impact of Combining Neutrophil-to-Lymphocyte Ratio with Sarcopenia for Improved Discrimination of Progression-Free Survival in Patients with Colorectal Cancer

**DOI:** 10.3390/jcm11020431

**Published:** 2022-01-15

**Authors:** Su Young Lee, Eric Chung, Eun-Suk Cho, Jae-Hoon Lee, Eun Jung Park, Su-Jin Shin, Seung Hyuk Baik, Kang Young Lee, Jeonghyun Kang

**Affiliations:** 1 Department of Surgery, Gangnam Severance Hospital, Yonsei University College of Medicine, 211 Eonju-ro, Gangnam-gu, Seoul 06273, Korea; lsy54312@gmail.com (S.Y.L.); camp79@yuhs.ac (E.J.P.); whitenoja@yuhs.ac (S.H.B.); 2 Department of Anesthesiology, Indiana University School of Medicine, Indianapolis, IN 46202, USA; erichyunchung@gmail.com; 3 Department of Radiology, Gangnam Severance Hospital, Yonsei University College of Medicine, 211 Eonju-ro, Gangnam-gu, Seoul 06273, Korea; JJONDOL@yuhs.ac; 4 Department of Nuclear Medicine, Gangnam Severance Hospital, Yonsei University College of Medicine, 211 Eonju-ro, Gangnam-gu, Seoul 06273, Korea; docnuke@yuhs.ac; 5 Department of Pathology, Gangnam Severance Hospital, Yonsei University College of Medicine, 211 Eonju-ro, Gangnam-gu, Seoul 06273, Korea; CHARM@yuhs.ac; 6 Department of Surgery, Severance Hospital, Yonsei University College of Medicine, 50-1 Yonsei-ro, Seodae-mun-gu, Seoul 03722, Korea; kylee117@yuhs.ac

**Keywords:** sarcopenia, NLR, colorectal cancer, survival, iAUC

## Abstract

This study aimed to evaluate the clinical impact of combined sarcopenia and inflammation classification (CSIC) in patients with colorectal cancer (CRC). The skeletal muscle index (SMI) and neutrophil-to-lymphocyte ratio (NLR) were measured in 1270 patients who underwent surgery between January 2005 and April 2014. A Cox proportional hazards model was used to evaluate the correlation of sarcopenia, NLR, and CSIC, with progression-free survival (PFS). The integrated area under the curve (iAUC) was used to compare the discriminatory performance of each model. Using the cut-off values for SMI suggested by Martin et al. and for an NLR of 2.26, the CSIC was defined as follows: nonsarcopenia with low NLR (group 1), nonsarcopenia with high NLR (group 2), sarcopenia with low NLR (group 3), and sarcopenia with high NLR (group 4). Sarcopenia alone was not statistically significant. Multivariate analysis identified that CSIC (group 4 vs. group 1; hazard ratio (HR), 1.726; 95% CI, 1.130–2.634; *p* = 0.011) and NLR (HR, 1.600; 95% CI, 1.203–2.128; *p* = 0.001) were independently associated with PFS. The CSIC improved the prediction accuracy of PFS compared with NLR (iAUC mean difference = 0.011; 95% CI, 0.0018–0.028). In conclusion, the combination of sarcopenia and NLR could improve prognostic accuracy, and thus compensate for the limitation of sarcopenia.

## 1. Introduction

In 2020, 1.9 million cases of CRC were estimated worldwide [1]. In particular, South Korea had the second highest incidence rate of CRC in the world, as of 2018 [2]. Considering this increasing prevalence, accurate prediction of prognosis in patients with CRC after treatment is now essential.

Traditionally, tumor-nodes-metastases (TNM) staging has been the most common and simple index for prediction of survival in cancer patients. Despite having its own clinical significance, researchers have reported limitations of staging in estimating patient survival, and recently, a modified staging system has been proposed to provide better prognostic discrimination and stratification in patients with nonmetastatic stage I–III CRC [3]. For similar reasons, many studies have been conducted to overcome such shortcomings in patients with CRC by focusing on host and environmental factors; therefore, systemic inflammation and body composition have been studied as indicators that reflect the host response to the tumor [4,5].

Neutrophil-to-lymphocyte ratio (NLR) was regarded as one of the simple serum markers that can reflect the systemic status of the host. The prognostic impact has been intensively investigated in various cancer patients [6,7,8,9].

In addition, low skeletal muscle index (SMI), a key feature of sarcopenia, has been known to be associated with poor oncologic outcomes, even in patients with CRC [5,10,11]. However, despite being validated by various studies and most other studies adopting cut-off values derived from previous pivotal studies, a number of studies have shown that sarcopenia is not related to survival in patients with CRC [12,13,14]. The reason for this discordance across studies has not been clearly proven; however, differences in body composition, including muscle mass, across different ethnicities might be one possible reason [15].

The clinical significance of the combination of systemic inflammation and sarcopenia is limited. It is still unclear whether a composite of sarcopenia and NLR could further enrich stratification power and thus overcome the limitation of the previously defined sarcopenia in patients with CRC. Thus, this study aimed to develop a combined classification using sarcopenia and NLR, and to compare its clinical usefulness with that of sarcopenia or NLR alone in patients with CRC.

## 2. Materials and Methods

### 2.1. Patients

Patients with CRC who were treated with surgery with/without chemotherapy between January 2005 and April 2014 at the Gangnam Severance Hospital of Yonsei University College of Medicine were initially selected. The inclusion criteria were as follows: (1) patients from whom we could extract computed tomography (CT) scan results and height information before surgery; (2) patients capable of undergoing muscle measurement to diagnose sarcopenia; and (3) patients who underwent surgery within 60 days of the CT scan. The exclusion criteria were as follows: (1) nonepithelial tumors such as neuroendocrine cell tumor or gastrointestinal stromal tumor; (2) patients with hereditary CRC or inflammatory bowel disease; (3) patents who underwent emergent surgery; (4) dual cancers; (5) tumors located in the appendix or anus; (6) patients with missing staging information; and (7) patients whose NLR tests were not performed 31 days before surgery or who had missing NLR values (Supplementary Materials Figure S4).

This study was approved by the ethical committee of our hospital. The need for informed consent was waived in this retrospective study.

### 2.2. Follow-Up after Surgery

Outpatient clinic visits were recommended to all patients every 3–6 months for up to 5 years. Carcinoembryonic antigen (CEA) levels were measured frequently during the follow-up visits. Abdominopelvic and/or chest CT scans were performed at an average interval of 6 months. A ^18^F-fluorodeoxyglucose positron emission tomography (PET) scan was considered when tumor recurrence was suspected or according to the surgeon’s discretion. A colonoscopy was indicated at the postoperative 1, 3 and 5 years. However, the time interval was also adjustable according to the detection of large size polys during follow up periods.

The median follow-up period was 91 (interquartile range, 67–115) months.

### 2.3. Measurement of SMI, NLR, and Combined Sarcopenia, and the Inflammation Classification (CSIC)

The SMI was calculated by dividing the skeletal muscle area (cm^2^) at the L3 level by the square of the height (m^2^). In our study, the cut-off values of SMI were adopted from a previous pivotal study by Martin et al. (Martin criteria), and were 43 cm^2^/m^2^ for men with body mass index (BMI) < 25 kg/m^2^, 53 cm^2^/m^2^ for men with BMI ≥ 25 kg/m^2^, and 41 cm^2^/m^2^ for all women [16]. In the univariate analysis, we also evaluated sarcopenia as defined by the sex-specific cut-off values of SMI described in a study by Prado and colleagues (Prado criteria), which were 52.4 cm^2^/m^2^ for men and 38.5 cm^2^/m^2^ for women [17].

NLR values were extracted from the blood sample data of the enrolled patients. The value that produced the largest χ^2^ on the Mantel–Cox test was chosen as the optimal cut-off value using X-tile program [18].

To further explore the prognostic value of the combination of SMI and NLR, we proposed a combined sarcopenia and inflammation classification (CSIC) by combining the Martin criteria and NLR. In the following survival analyses, patients were categorized into four groups based on the CSIC: nonsarcopenia with low NLR (group 1); nonsarcopenia with high NLR (group 2); sarcopenia with low NLR (group 3); and sarcopenia with high NLR (group 4).

### 2.4. Statistical Analyses

Categorical variables were analyzed using the chi-squared test and continuous variables were analyzed using the Kruskal–Wallis test. The time between the date of surgery and either the date of recurrence, last follow-up or death of any cause, was used to define progression-free survival (PFS).

The Kaplan–Meier method was used to estimate PFS and was statistically compared with the log-rank test. The Cox proportional hazards model was used to determine the predictive factors associated with PFS, and to estimate hazard ratios (HRs) and 95% confidence intervals (CIs). Variables with *p* < 0.10 in the univariate analysis were selected and entered into a multivariate model with backward conditional elimination. Considering multicollinearity, CSIC and NLR were evaluated in separate sets of analyses to evaluate their impact independently, without the effect of the other. Time-dependent receiver operating characteristic (ROC) curves were used to compare prognostic abilities of respective variables. Time-dependent ROC-curve analysis reflects the changing disease status and marker values according to time, which is more reasonable when compared with the classical ROC-curve analysis. The prognostic accuracy between the CSIC and NLR was compared by calculating the integrated AUC (iAUC) difference of the ROC.

A two-sided *p*-value of <0.05 was considered statistically significant. R version 3.6.3 (R-project, Institute for Statistics and Mathematics, Vienna, Austria) was used for statistical analyses.

## 3. Results

### 3.1. Demographic Characteristics

A total of 1270 patients who underwent surgery with or without chemotherapy for CRC were included in the study. The baseline demographics of the patients are compared according to sarcopenia, as defined by Martin et al., in Table 1. Sex, age, BMI, tumor location, tumor size, and status of receiving chemotherapy were independently associated with sarcopenia, whereas CEA, histologic grade, lymphovascular invasion (LVI), complications, lymph node numbers, and the American Joint Committee on Cancer (AJCC) stage were not. The mean NLR value was significantly higher in the sarcopenia group than in the nonsarcopenia group (mean ± standard deviation, 3.3 ± 2.8 vs. 2.8 ± 2.3; *p* = 0.012).

### 3.2. Survival Analyses Based on Sarcopenia and NLR According to Various Criteria

**Figure 1:**
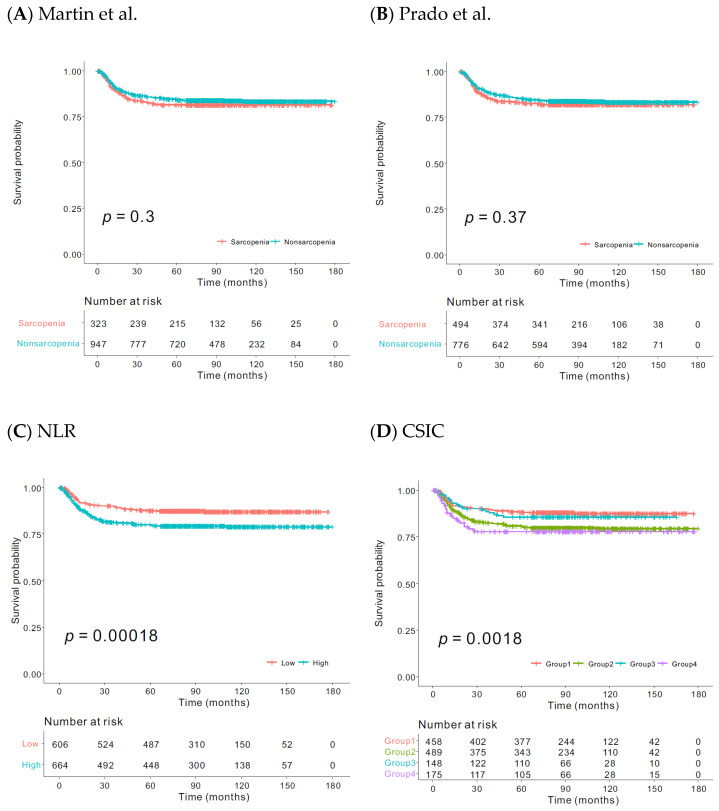
Kaplan–Meier curves of progression-free survival (PFS) in the groups, based on sarcopenia according to (**A**) the criteria by Martin et al., (**B**) criteria by Prado et al., (**C**) neutrophil-to-lymphocyte ratio (NLR), and (**D**) combined sarcopenia and inflammation classification (CSIC).

The optimal cut-off value of NLR to discriminate PFS in the overall group was determined to be the value that produced the largest χ2 in the Mantel-Cox test, which was 2.26 (Supplementary Materials Figure S1). On the Kaplan–Meier survival curve, sarcopenia alone showed no significant association with PFS. In contrast, survival analysis based on the CSIC showed significant differences in PFS among the four groups on the Kaplan–Meier survival curve (*p* = 0.0018) (Figure 1). When we divided patients into colon and rectal cancer, and provided Kaplan–Meier survival curves according to the tumor location (Supplementary Materials Figures S2 and S3), we could infer that the statistical significance of CSIC may be stronger in rectal cancer than that of the colon cancer.

### 3.3. Factors Associated with Survival in Patients with CRC

In univariate analysis, BMI, CEA, tumor size, histologic grade, LVI, stage, complications, chemotherapy, NLR and CSIC were identified as significant factors (Table 2). These variables except CSIC were entered into a multivariate analysis in the first stage (Table 3).

CSIC (group 2 vs. group 1: HR, 1.599, 95% CI, 1.146–2.231; and group 4 vs. group 1: HR, 1.726; 95% CI, 1.130–2.634; *p* = 0.011) was proven to be an independent risk factor for PFS. In the second stage, we entered the NLR, instead of the CSIC, into a multivariate model (Table 3). The results showed that NLR (high vs. low: HR, 1.600; 95% CI, 1.203–2.128; *p* = 0.001) was independently associated with PFS.

### 3.4. Improved Discrimination Capacity for Prognosis by CSIC Compared to NLR Only

**Figure 2:**
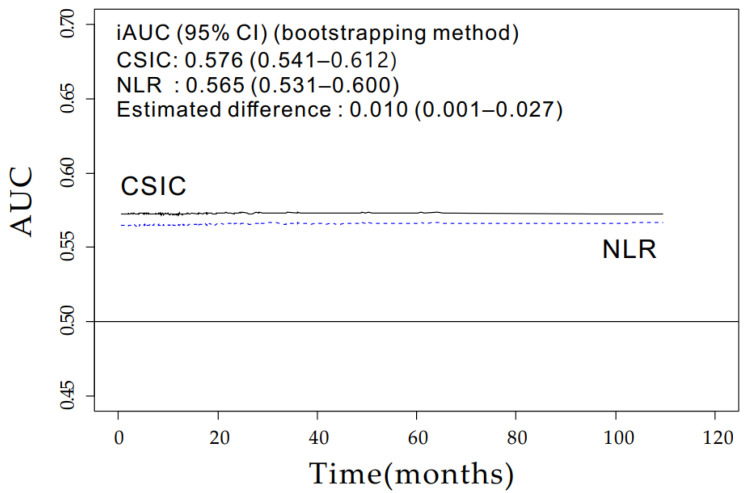
Time-dependent area under the curve (AUC) plot for comparing the prognostic accuracy of the combined sarcopenia and inflammation classification (CSIC; solid line) and neutrophil-to-lymphocyte ratio (NLR; dashed line). Bootstrap iAUC mean values of CSIC and NLR are 0.576 (95% CI, 0.541–0.612) and 0.565 (95% CI, 0.531–0.600), respectively. The increased integrated AUC (iAUC) of CSIC when compared with NLR represents the superior prognostic accuracy of CSIC (bootstrap iAUC mean difference = 0.010; 95% CI, 0.001–0.027).

The time-dependent ROC curve of the CSIC was superior to that of the NLR (bootstrap iAUC mean difference = 0.010; 95% CI, 0.001–0.027) throughout the observation period. The addition of sarcopenia to the NLR (CSIC) significantly improved the model discrimination capacity (Figure 2).

## 4. Discussion

Our study demonstrated that the most commonly used criteria for defining sarcopenia could not adequately predict the PFS of our patients. The CSIC, an index that combines SMI and systemic inflammatory marker, is clinically meaningful for better discrimination of survival in patients with CRC.

As it is known that the long-term survival of patients with CRC is affected by patient-related factors, the effect of body composition has recently been brought to the spotlight [5,19]. Skeletal muscle area can be relatively easily measured using single-slice CT images, and is a parameter that could represent the total amount of muscle in humans [20,21]. Many studies have reported that sarcopenia, defined using the criteria presented by Martin et al. or Prado et al., worsens long-term survival in patients with CRC [5,10,11]. On the other hand, there are still shortcomings in applying the previous pivotal definitions of sarcopenia to clinical use. Many studies have shown no difference in survival according to the criteria of sarcopenia defined by Martin et al. and/or Prado et al. [12,13,14,19,22]. It is difficult to explain, in brief, why the clinical significance of sarcopenia differs in each study. Due to the difference in the basic mean value of muscle according to race or patient group, a few reports have mentioned the requirement for proper criteria for Asian countries in a straightforward manner [23,24]. Notably, it appears that the previously well-known criteria for sarcopenia were derived from multiple types of different cancer patients of mostly advanced status, and the diversity of stage, type of cancer, and patient characteristics in other cohorts could also make a difference. For example, the criteria suggested by Prado et al. were obtained by risk stratification in obese patients (BMI ≥ 30 kg/m^2^) with solid tumors of the respiratory or gastrointestinal tracts. Therefore, it was suggested that the sarcopenia index defined in a specific situation may be too limited for general use in all other patients. In our study using a relatively large cohort of patients with CRC, it was also shown that sarcopenia defined using well-known criteria alone could not predict PFS effectively (Martin’s criteria, *p* = 0.305; Prado’s criteria, *p* = 0.373 in the univariate Cox proportional hazards model). These observations emphasize the need for an improved prognosticator to overcome these limitations.

Systemic inflammation has long been considered an essential prognosticator in patients with CRC, and is complementary to sarcopenia [4,25]. Systemic inflammation is related to functional and immunological decline in patients, although the exact pathophysiology needs to be further elucidated. NLR is a representative marker of systemic inflammation, and is associated with elevated levels of various proinflammatory cytokines and chemokines such as IL-6, IL-7, and CXCL8 in patients with CRC [25]. Such cytokines can inhibit protein synthesis, resulting in the loss of muscle mass, and IL-6, in particular, is an essential factor in the progression of cancer-related cachexia [26]. The catabolism of skeletal muscle can also inversely potentiate systemic inflammation by increasing plasma glutamine levels. Glutamine is one of the major energy sources utilized by lymphocytes, and mainly originates from skeletal muscle to enter the circulation [27].

These findings imply that systemic inflammation and sarcopenia are mutually inducible. This close relationship may exaggerate and compensate for the prognostic power of systemic inflammation and sarcopenia. Previous studies have suggested that prognostic ability could be improved by combining systemic inflammatory markers with the sarcopenia index rather than by using the sarcopenia criteria alone. Feliciano et al. demonstrated that the overall and CRC-related risk of death increased by more than two times in nonmetastatic CRC patients with NLR >3 having sarcopenia at the same time [11]. Sarcopenia was reported to be a powerful predictor of survival in the high NLR group alone, and not in the low NLR group of patients with esophageal cancer [28]. In our study, we established the CSIC by combining preoperatively measured NLR and sarcopenia, which could classify patients in more detail. According to the multivariate analysis, groups 2 (nonsarcopenia with high NLR) and 4 (sarcopenia with high NLR) showed a markedly increased risk of recurrence (HR, 1.599 and 1.726; *p* = 0.005 and *p* = 0.011, respectively). However, patients with sarcopenia and low NLR showed a similar recurrence rate to patients with no sarcopenia and low NLR (HR, 1.076; *p* = 0.779). This result indicated that systemic inflammation may be a more prominent indicator for estimating disease recurrence than sarcopenia alone.

It is still debatable if sarcopenia represents either chronological deterioration of muscle or disease-specific changes in patients with cancer. In a recent meta-analysis by our group, skeletal muscle radiodensity (SMD), which represents fat infiltration in skeletal muscle area and is regarded as another type of sarcopenic status, was proven to be a significant prognostic factor with respect to overall survival [29]. Nevertheless, the clinical utility of SMD in disease-free survival (DFS) is not evident, showing no difference in DFS between the low and high SMD groups. Considering these previous observations, our results need to be interpreted in a multifaceted manner. First, sarcopenia itself may be more related to age-related changes in skeletal muscle rather than reflecting an advanced disease stage. Another possibility remains that the criteria for sarcopenia need to be applied appropriately, with consideration of different patient characteristics. Our findings also suggest that combining systemic inflammation and sarcopenia criteria could overcome the limitation of sarcopenia alone while trying to estimate the recurrence potential. Nonetheless, sarcopenia still appears to have an incremental effect in predicting disease recurrence, as evidenced by the fact that the CSIC showed better discriminatory performance than NLR when evaluated by the iAUC, although our assumption needs to be validated in other cohorts.

The limitations of our study are as follows. This study was a retrospective and single-institution-based study; thus, selection bias was inevitable. Another is that the cut-off value of NLR in the study was a value determined particularly in our cohort, as the optimal cut-off values for NLR are different across studies. Further analysis is needed to determine if the CSIC determined in this way can be applied to other cohorts.

## 5. Conclusions

Our study showed that conventionally used definitions of sarcopenia were not significant factors with respect to PFS, and that combined inflammation with sarcopenia could be an alternative and practical way to overcome such limitations.

## Figures and Tables

**Table 1:** Patient characteristics according to sarcopenia defined by Martin (*n* = 1270).

		Sarcopenia (*n* = 323) N (%)	Nonsarcopenia (*n* = 947) N (%)	*p*
Sex	Female	180 (55.7)	332 (35.1)	<0.001
	Male	143 (44.3)	615 (64.9)	
Age (years)	<70	210 (65.0)	685 (72.3)	0.016
	≥70	113 (35.0)	262 (27.7)	
BMI (kg/m^2^)	<25	266 (82.4)	653 (69.0)	<0.001
	≥25	57 (17.6)	294 (31.0)	
CEA (ng/mL)	<5	202 (62.5)	603 (63.7)	0.926
	≥5	106 (32.8)	303 (32.0)	
	Not available	15 (4.6)	41 (4.3)	
Tumor location	Right colon	90 (27.9)	217 (22.9)	0.037
	Left colon	142 (44.0)	394 (41.6)	
	Rectum	91 (28.2)	336 (35.5)	
Tumor size (cm)	<5	172 (53.3)	600 (63.4)	0.002
	≥5	151 (46.7)	347 (36.6)	
Histologic grade	G1 and G2	294 (91.0)	872 (92.1)	0.260
	G3	10 (3.1)	38 (4.0)	
	Mucinous and SRC	19 (5.9)	37 (3.9)	
LVI	Absent	212 (65.6)	642 (67.8)	0.775
	Present	79 (24.5)	217 (22.9)	
	Not available	32 (9.9)	88 (9.3)	
Complications	No	242 (74.9)	715 (75.5)	0.894
	Yes	81 (25.1)	232 (24.5)	
LN numbers	<12	46 (14.2)	166 (17.5)	0.200
	≥12	277 (85.8)	781 (82.5)	
AJCC Stage	I and II	161 (49.8)	497 (52.5)	0.404
	III	119 (36.8)	349 (36.9)	
	IV	43 (13.3)	101 (10.7)	
Chemotherapy	Yes	147 (45.5)	358 (37.8)	0.017
	No	176 (54.5)	589 (62.2)	
NLR	Mean (SD)	3.3 (2.8)	2.8 (2.3)	0.012

SD: Standard deviation, BMI: body mass index, CEA: carcinoembryonic antigen, G1: well differentiated (low grade), G2: moderately differentiated (intermediate grade), G3: poorly differentiated (high grade), SRC: signet ring cell, LVI: lymphovascular invasion, LN: lymph node, NLR: neutrophil-to-lymphocyte ratio, AJCC: American Joint Committee on Cancer.

**Table 2:** Univariate analysis of factors associated with progression-free survival (*n* = 1270).

Variables	Parameters	Number of Events (%)	HR (95% CI)	*p*
Sex	Female	86/512 (16.7)	1	
	Male	120/758 (15.8)	0.942 (0.714–1.243)	0.674
Age (years)	<70	151/895 (16.8)	1	
	≥70	55/375 (14.6)	0.935 (0.687–1.274)	0.672
BMI (kg/m^2^)	<25	163/919 (17.7)	1	
	≥25	43/351 (12.2)	0.647 (0.462–0.906)	0.011
CEA (ng/mL)	<5	102/805 (12.6)	1	
	≥5	97/409 (23.7)	2.117 (1.603–2.797)	<0.001
	Not available	7/56 (12.5)	0.971 (0.451–2.089)	0.941
Tumor location	Right Colon	43/307 (14.0)	1	
	Left Colon	85/536 (15.8)	1.122 (0.777–1.620)	0.537
	Rectum	78/427 (18.2)	1.314 (0.905–1.907)	0.150
Tumor size (cm)	<5	115/772 (14.8)	1	
	≥5	91/498 (18.2)	1.323 (1.005–1.742)	0.046
Histologic grade	G1 and G2	179/1166 (15.3)	1	
	G3	11/48 (22.9)	1.713 (0.931–3.149)	0.083
	Mucinous and SRC	16/56 (28.5)	2.039 (1.222–3.401)	0.006
LVI	Absent	97/854 (11.3)	1	
	Present	86/296 (29.0)	3.108 (2.324–4.157)	<0.001
	Not available	23/120 (19.1)	1.747 (1.109–2.753)	0.016
LN numbers	<12	40/212 (18.8)	1	
	≥12	166/1058 (15.6)	0.822 (0.582–1.162)	0.268
AJCC Stage	I and II	50/658 (7.5)	1	
	III	103/468 (22.0)	3.283 (2.341–4.602)	<0.001
	IV	53/144 (36.8)	7.311 (4.958–10.782)	<0.001
Complications	No	145/957 (15.1)	1	
	Yes	61/313 (19.4)	1.458 (1.081–1.966)	0.013
Chemotherapy	No	65/505 (12.8)	1	
	Yes	141/765 (18.4)	1.346 (1.003–1.805)	0.047
NLR	Low (<2.26)	75/606 (12.3)	1	
	High (≥2.26)	131/664 (19.7)	1.71 (1.287–2.271)	<0.001
Sarcopenia (Martin)	Yes	56/323 (17.3)	1	
	No	150/947 (15.8)	0.851 (0.626–1.157)	0.305
Sarcopenia (Prado)	Yes	84/494 (17.0)	1	
	No	122/776 (15.7)	0.881 (0.667–0.373)	0.373
CSIC	Group 1	55/458 (12.0)	1	
	Group 2	95/489 (19.4)	1.713 (1.229–2.388)	0.001
	Group 3	20/148 (13.5)	1.176 (0.705–1.962)	0.534
	Group 4	36/175 (20.5)	1.989 (1.306–3.028)	0.001

HR: Hazard ratio, CI: confidence interval, BMI: body mass index, CEA: carcinoembryonic antigen, G1: well differentiated (low grade), G2: moderately differentiated (intermediate grade), G3: poorly differentiated (high grade), SRC: signet ring cell, LVI: lymphovascular invasion, LN: lymph node, NLR: neutrophil-to-lymphocyte ratio, CSIC: combined sarcopenia and inflammation classification, AJCC: American Joint Committee on Cancer.

**Table 3:** Multivariate analysis of factors associated with progression-free survival.

		Model 1		Model 2	
		HR (95% CI)	*p*	HR (95% CI)	*p*
BMI (kg/m^2^)	<25	1		1	
	≥25	0.723 (0.514–1.015)	0.061	0.718 (0.512–1.008)	0.055
LVI	Absent	1		1	
	Present	1.911 (1.407–2.595)	<0.001	1.915 (1.411–2.600)	<0.001
	Not available	1.883 (1.191–2.974)	0.006	1.879 (1.189–2.968)	0.006
AJCC Stage	I and II	1		1	
	III	2.854 (2.003–4.067)	<0.001	2.857 (2.006–4.069)	<0.001
	IV	5.731 (3.811–8.617)	<0.001	5.733 (3.814–8.618)	<0.001
CSIC	Group 1	1			
	Group 2	1.599 (1.146–2.231)	0.005		
	Group 3	1.076 (0.643–1.799)	0.779		
	Group 4	1.726 (1.130–2.634)	0.011		
NLR	Low (<2.26)			1	
	High (≥2.26)			1.600 (1.203–2.128)	0.001

HR: Hazard ratio, CI: confidence interval, BMI: body mass index, LVI: lymphovascular invasion, NLR: neutrophil-to-lymphocyte ratio, CSIC: combined sarcopenia and inflammation classification, AJCC: American Joint Committee on Cancer.

## Data Availability

The datasets generated and/or analyzed during the current study are available from the corresponding author on reasonable request pending the approval of the institution(s) and trial/study investigators who contributed to the dataset.

## Supplementary Materials

The following are available online at https://www.mdpi.com/article/10.3390/jcm11020431/s1, Supplementary Materials Figure S1: Defining Cut-off value of neutrophil-to-lymphocyte ratio (NLR) to discriminate PFS in overall group, Supplementary Materials Figure S2: Kaplan–Meier survival curves according to the sarcopenia and inflammatory markers in colon cancer patients (*n* = 843), Supplementary Materials Figure S3: Kaplan–Meier survival curves according to the sarcopenia and inflammatory markers in rectal cancer patients (*n* = 427), Supplementary Materials Figure S4: Inclusion Flow.
