# Supplementary material for: The Clinical Impact of Combining Neutrophil-to-Lymphocyte Ratio with Sarcopenia for Improved Discrimination of Progression-Free Survival in Patients with Colorectal Cancer
Source: J Clin Med. 2022 Jan 15;11(2):431. doi: 10.3390/jcm11020431 (PMC8780466; doi:10.3390/jcm11020431)

Supplementary File

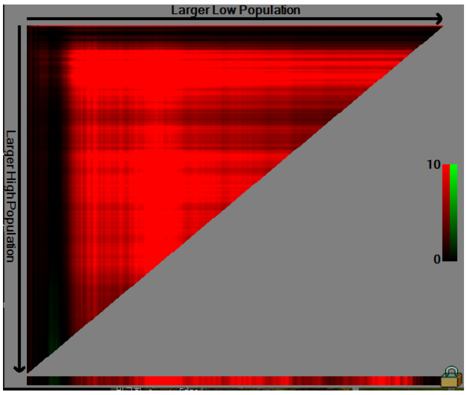

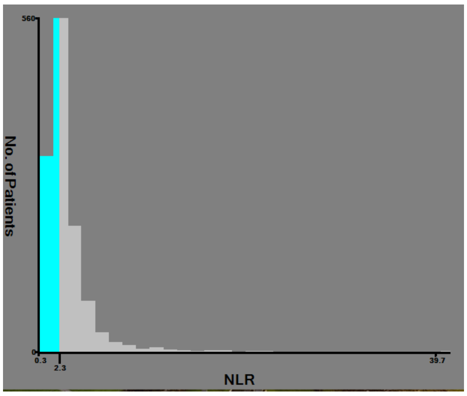

Supplementary Figure S1. Defining Cut-off value of neutrophil-to-lymphocyte ratio (NLR) to discriminate PFS in overall group

The points of the variable coloration of the X-tile plot represent the strength of the association at each division ranging from low (dark, black) to high (bright, red, or green). Red and green represent an inverse and direct association between the expression levels and survival of the variables respectively. The optimal cut-off value was defined as the values that produced the largest $\chi2$ in the Mantel-Cox test, and this was set as 2.26.

Supplementary Figure S2. Kaplan-Meier survival curves according to the sarcopenia and inflammatory markers in colon cancer patients (n=843)

(A) Martin et al.

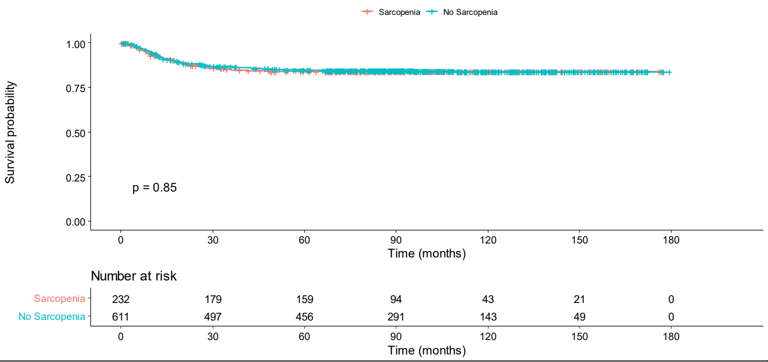

(B) Prado et al.

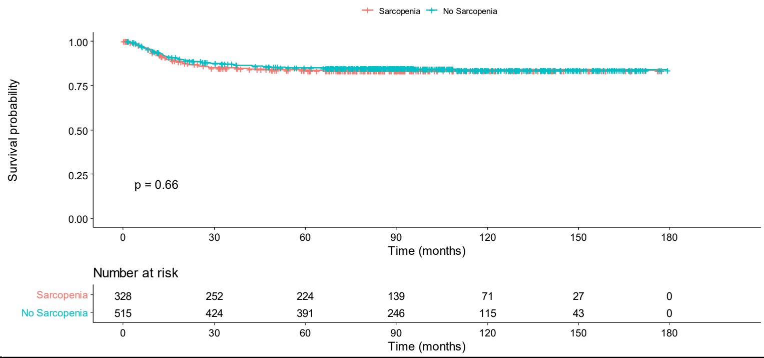

(C) NLR

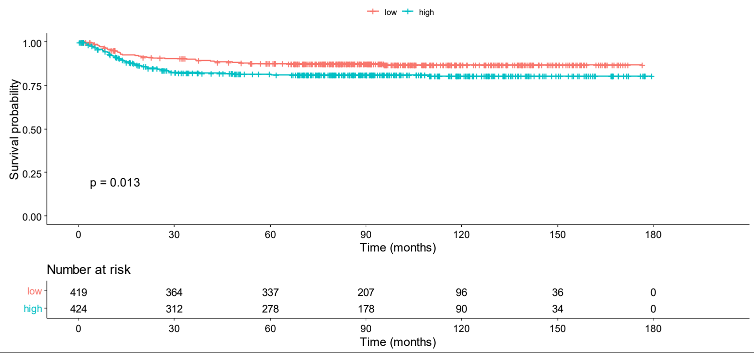

(D) CSIC

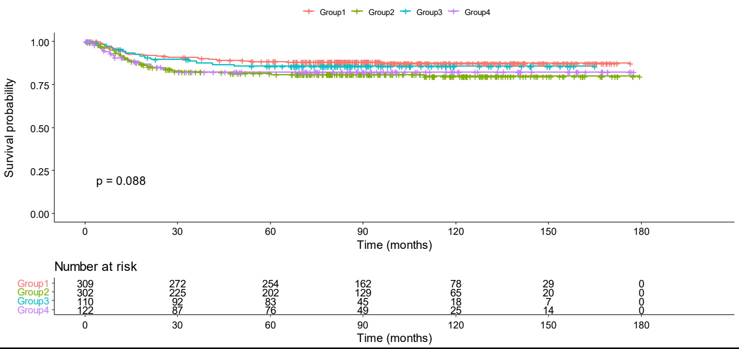

Supplementary Figure S3. Kaplan-Meier survival curves according to the sarcopenia and inflammatory markers in rectal cancer patients (n=427)

(A) Martin et al.

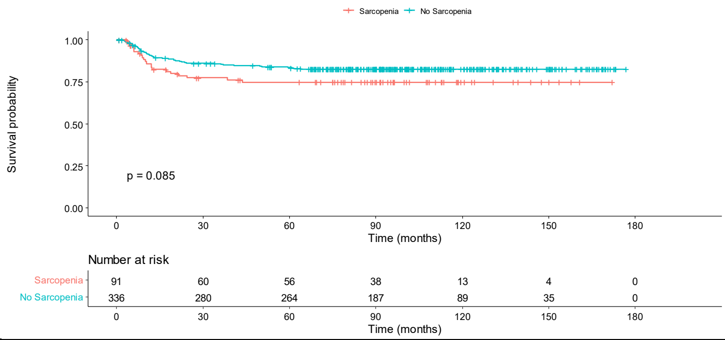

(B) Prado et al.

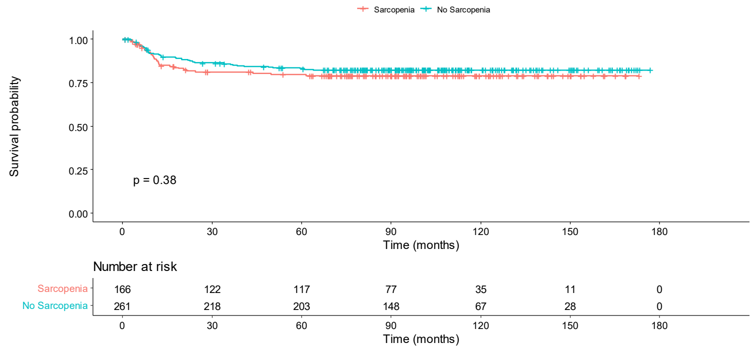

(C) NLR

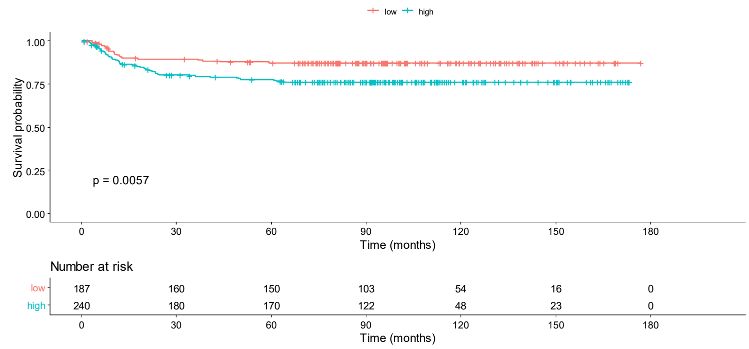

(D) CSIC

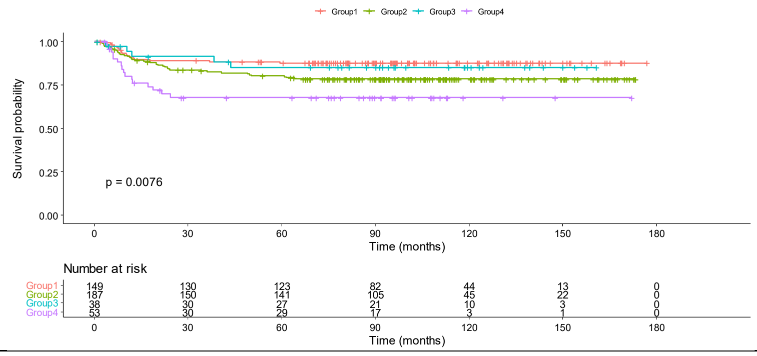

Supplementary Figure S4. Inclusion flow

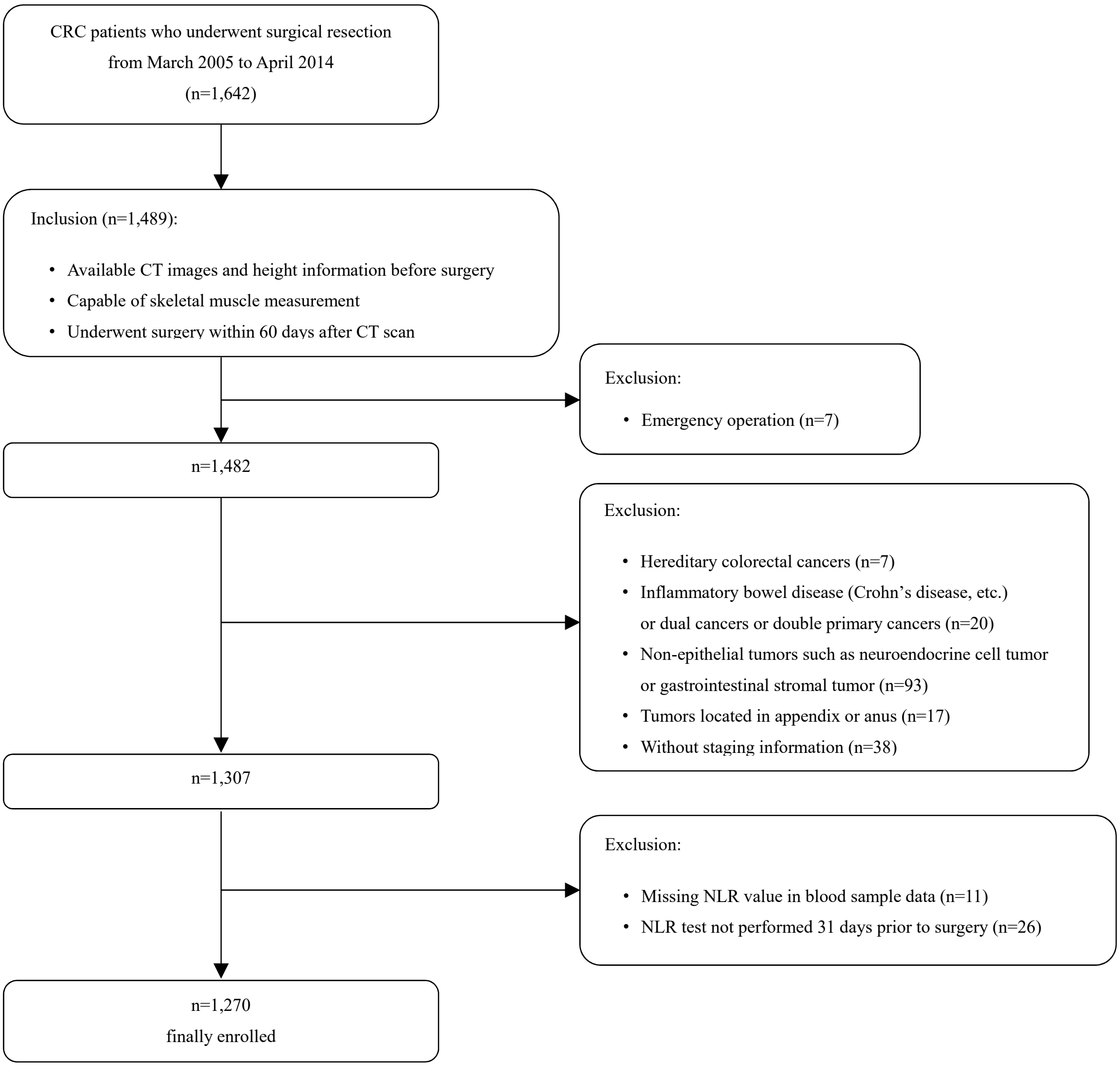

Supplement: Supplementary file 1 [file jcm-11-00431-s001.zip › jcm-1447673-supplementary.pdf]
